# Vivir con psicofármacos: un estudio fotovoz comunitario en personas con alta adherencia al tratamiento en el sureste de España

**DOI:** 10.18294/sc.2024.5090

**Published:** 2024-10-29

**Authors:** Francisco Martínez-Granados, Erica Briones-Vozmediano, Elena Ronda

**Affiliations:** 1 Doctor en Ciencias de la Salud. Jefe de Unidad de Farmacia. Centro psiquiátrico socio-asistencial Doctor Esquerdo, Alicante, España. pacomartinezgranados@gmail.com Centro psiquiátrico socio-asistencial Doctor Esquerdo Centro psiquiátrico socio-asistencial Doctor Esquerdo Alicante Spain pacomartinezgranados@gmail.com; 2 Autora de correspondencia. Doctora en Salud Pública. Profesora Agregada en Departamento de Enfermería y Fisioterapia, Facultad de Enfermería y Fisioterapia, Universidad de Lleida. Grupo de Investigación en Salud, Educación y Cultura (GESEC), Universidad de Lleida. Grup de Recerca en Cures en Salut (GRECS), Institut de Recerca Biomèdica (IRB), Lleida, España. erica.briones@udl.cat Universitat de Lleida Departamento de Enfermería y Fisioterapia Facultad de Enfermería y Fisioterapia Universidad de Lleida Lleida Spain erica.briones@udl.cat; Investigación en Salud, Educación y Cultura (GESEC) Universidad de Lleida; Grup de Recerca en Cures en Salut (GRECS) Institut de Recerca Biomèdica (IRB); 3 Doctora en Medicina. Catedrática de Medicina Preventiva y Salud Pública, Área de Medicina Preventiva y Salud Pública, Universidad de Alicante, Alicante, España. Área de Epidemiología y Salud Pública, Centro de Investigación Biomédica en Red (CIBERESP), Madrid, España. elena.ronda@ua.es Universidad de Alicante Área de Medicina Preventiva y Salud Pública Universidad de Alicante Alicante Spain elena.ronda@ua.es; Centro de Investigación Biomédica en Red (CIBERESP)

**Keywords:** Salud Mental, Psicofármacos, Adherencia a los Medicamentos, Servicios Comunitarios de Salud Mental, España, Mental Health, Psychotropic Drugs, Medication Adherence, Community Mental Health Services, Spain

## Abstract

La adherencia al tratamiento psicofarmacológico consiste esencialmente en un proceso de construcción de significado. El objetivo de este estudio fue explorar la experiencia de personas bajo tratamiento psiquiátrico crónico desde la perspectiva de los pacientes. En 2018, desde la metodología de fotovoz, se realizaron cuatro sesiones, en las que participaron 11 personas de una escuela comunitaria de un barrio de alto riesgo de exclusión social de una ciudad del sureste de España, algunas de las cuales eran, además, usuarias en un centro de rehabilitación e integración social y de un centro de día. Las personas participantes realizaron y escogieron 41 fotografías con la premisa: “Fotografía tu vivencia en torno a la salud y la medicación”. Se realizó una categorización de las narrativas emergentes en una sesión dialógica entre participantes e investigadores que fue grabada para su posterior transcripción y análisis. Las experiencias pudieron desglosarse en dos grandes núcleos simbólicos que buscan dotar de sentidos terapéuticos a la medicación: uno de conflictividad y otro motivacional.

## Abstract (en)

Adherence to psychopharmacological treatment essentially involves a process of meaning-making. The objective of this study was to explore the experience of people undergoing chronic psychiatric treatment from the patients’ perspective. In 2018, using the photovoice methodology, four sessions were conducted with the participation of 11 individuals from a community school in a socially marginalized neighborhood of a southeastern Spanish city, some of whom were also users of a social rehabilitation and integration center and a day center. Participants took and selected 41 photographs under the premise: “Photograph your experience regarding health and medication.” A categorization of the emerging narratives was carried out during a dialogical session between participants and researchers, which was recorded for subsequent transcription and analysis. The experiences were divided into two major symbolic themes that seek to give therapeutic meaning to medication: one of conflict and the other of motivation.

## INTRODUCCIÓN

La adherencia al tratamiento psiquiátrico es cuantitativamente baja, entre el 35% y el 60%^1^, y cualitativamente conflictiva^2^. Esta adherencia problemática es multifactorial^3^^,^^4^ y no se resolvería con medidas psicoeducativas basadas en la provisión de información^5^^,^^6^. La conducta de adherirse al tratamiento forma parte de la esfera de la subjetividad^7^^,^^8^^,^^9^ y, en particular, depende de aspectos experienciales y culturales que acaban fraguando los sistemas de valores, significados, creencias y actitudes hacia la medicación^10^^,^^11^. La experiencia medicamentosa ha sido definida como “la experiencia subjetiva de tomar medicación a diario”^12^ y muestra una enorme influencia en aspectos claves que determinan el compromiso con la psicofarmacoterapia^13^. 

Desde la antropología se entiende que la acción humana está fuertemente determinada por “fuerzas simbólicas culturales”, es decir, por los significados que otorgamos a las cosas y hechos de la realidad^14^. Victor Turner y la Escuela de Manchester defienden que “cultura” es sinónimo de “pugna”, de *significados en conflicto*^15^^,^^16^. Stuart Hall defiende que el locus privilegiado donde tiene lugar esta pugna es el configurado por “prácticas significantes” (productoras de sentido) en las que puedan darse los intercambios dialógicos, simbólicos y, ante todo, significantes, que todo proceso multicultural necesita^17^.

Aun así, son escasos los estudios cualitativos que abordan la experiencia farmacoterapéutica con el objetivo de comprender la conflictividad que subyace a la toma de medicamentos psiquiátricos. Un estudio cualitativo en EEUU exploró la experiencia psicofarmacoterapéutica mostrando que consistía en un proceso de construcción de sentidos terapéuticos al que denominaron “aceptando la medicación”^18^; proceso que se hallaba co-implicado en una experiencia psiquiátrica y vital más amplia. La metodología que emplearon fue el fotovoz que, además de ser un método de investigación de acción participativa, es en sí misma una práctica significante con la capacidad de activar propedéutica y políticamente a las comunidades^19^^,^^20^. Según Wang, permite “que los profesionales de la salud e investigadores tengan la posibilidad de percibir el mundo desde la perspectiva de las personas que viven vidas diferentes con respecto a quienes tienen el control sobre el significado y las imágenes del mundo”^20^. Ha sido empleado preferentemente en colectivos estigmatizados y/o en grupos que, tanto por sus condiciones de salud (deterioro cognitivo, abuso de drogas, trastorno mental grave), como por preferencia y/o bagaje cultural, muestran un mayor desarrollo de su capacidad expresiva a través de imágenes^19^^,^^21^^,^^22^^,^^23^^,^^24^^,^^25^. Además, algunos de estos colectivos son precisamente los más vulnerables tanto epistémicamente^26^, como en vulneración de derechos humanos^27^.

En la actualidad, los llamados “Estudios Locos” (*Mad studies*) proponen rechazar al modelo biomédico, poner en el centro la ética, los derechos, lo social y lo holístico (en detrimento de concepciones individualistas y psiquiatrizantes del sufrimiento humano), dar más valor a lo experiencial y al conocimiento en primera persona, movilizar colectivos y comunidades y promover su activación propedéutica y política con respecto a la salud mental de los pueblos^26^. Estos principios son esencialmente los mismos que los generados por la metodología de investigación acción-participativa de fotovoz.

En Brasil y en Cataluña ha tenido lugar en las últimas décadas una experiencia colectiva (que puede enmarcarse dentro del concepto de práctica significante en torno a la medicación psiquiátrica) en lo que se ha denominado el “modelo de gestión autónoma de la medicación”^28^ y en la que se pone en práctica una decolonización del concepto de autonomía de corte individualista hacia una concepción performativa, propedéutica y político-democrática de las comunidades con el fin de equilibrar los desequilibrios de poder instaurados por el paradigma biomédico^27^. De esta experiencia surge la “Guía para la gestión colaborativa de la medicación en salud mental”^29^, que ofrece información relevante, recursos y herramientas para llevar a cabo una práctica significante y armar a los colectivos para que puedan implicarse activamente en el proceso de toma de decisiones acerca de su medicación y otros determinantes sociales de la salud mental. 

Comprender, por tanto, la problemática de la adherencia como un proceso multicultural puede contribuir a la resolución ética de los conflictos que se producen a diario en nuestro entorno y que, con frecuencia, pasan por el recurso a prácticas violentas como la coacción, la contención mecánica o la medicación forzosa^30^^,^^31^^,^^32^, lo que conlleva una violación de los derechos humanos, tal y como lo denuncian desde el informe del Relator Especial de Naciones Unidas^27^, hasta los propios “supervivientes” del sistema de salud mental englobados en movimientos activistas como el del “Orgullo Loco”^32^. Con el fin de situar la ética en el centro de las prácticas en salud mental está justificado investigar en torno a la dimensión simbólica de la medicación psiquiátrica. El presente estudio tuvo el objetivo de explorar y analizar en profundidad las experiencias con la psicofarmacoterapia en personas en exclusión social que viven en la comunidad y que están comprometidas activamente con sus procesos de rehabilitación. 

## MÉTODOS

Este estudio fotovoz se realizó en una escuela comunitaria dentro de un barrio de alto riesgo de exclusión social de una ciudad del sureste de España en el año 2018. Algunos de las personas participantes eran, además, usuarias en un centro de rehabilitación e integración social y de un centro de día. 

Los participantes fueron reclutados de forma intencional. Los criterios de inclusión fueron que estuviesen en tratamiento con al menos un psicofármaco, durante al menos 12 meses. El único criterio de exclusión fue estar en fase aguda o subaguda (no haber tenido ningún ingreso hospitalario por descompensación psiquiátrica en el último año).

El trabajo de campo duró tres meses durante los que se realizaron cuatro sesiones con los participantes. En la primera sesión se les enseñó técnicas de expresión artística a través de la fotografía, se abordaron los aspectos éticos y se les dio la premisa *“fotografía tu vivencia en torno a la salud y la medicación*”. Las fotografías se hicieron con cámara réflex cedida por alguno de los centros o con los teléfonos móviles de los participantes. Se dividió a los grupos por sexo para minimizar un sesgo de género en la emergencia y exploración de las narrativas. Cada participante trajo como máximo cinco fotografías a la segunda sesión. La tercera sesión consistió en una dinámica grupal, en la que se propuso clasificar las fotografías según reflejasen aspectos positivos o negativos de sus vivencias.

Una vez que se valoró que las narrativas que emergieron de los dos grupos pequeños no iban a ser eclipsadas por un factor de sexo-género en una sesión de categorización común, se realizó la cuarta sesión de forma conjunta. El objetivo fue construir categorías sobre la base de los temas que emergieron en las sesiones dos y tres. Se expusieron todas las fotografías en una pizarra, de manera que pudieran ser movidas sin dificultad a lo largo de su superficie. A continuación, se continuaba con el proceso dialógico. La premisa fue reordenar las fotografías por dominios semánticos y relacionarlos entre sí. Al terminar, el investigador participó en la categorización proponiendo denominaciones en algunos casos, pero en todo momento se pedía la verificación del sentido originario a los participantes. Se priorizó la denominación de la categoría que emergió espontáneamente del grupo.

Tres meses después de la cuarta sesión, tuvo lugar una exposición fotográfica durante dos días en un centro cultural de la ciudad a la que se invitó a agentes claves que formaran parte de instituciones políticas o sanitarias y a un periodista. 

La cuarta sesión de categorización fue grabada en audio y transcrita en su totalidad. A continuación, el primer autor realizó el análisis, supervisado por otra de las autoras. Se dividió el texto transcrito en unidades de significado, fracciones del texto narrativo que contenían un sentido distinguible. El proceso de codificación consistió en condensar estas primeras unidades en *unidades de significado condensadas*, que contenían de manera sintetizada el significado asociado al texto, y asignar códigos abiertos emergentes. A partir de su agrupación surgieron las diferentes “Subcategorías” que luego fueron clasificadas dentro de cada una de las categorías finales que emergieron de forma participativa de la cuarta sesión (Tabla 1).

**Tabla 1:** Categorías y subcategorías emergentes del fotovoz. España, 2018.

Categoría	Subcategoría
1. Cambio radical de vida a partir de la medicación	1.1. Primeras experiencias con la medicación psiquiátrica: el acontecimiento radical
	1.2. Dependencia, cronificación, infertilidad: “La medicación no es un juego”
	1.3. La medicación como símbolo de la caída del paraíso
	1.4. ¿Cómo sería nuestra vida sin medicación?
	1.5. El impacto de la medicación en las formas y hábitos de vida
2. Cambios en la percepción, el pensamiento y/o el afecto	2.1. Cambios en el pensamiento y la percepción
	2.2. Medicación e identidad
	2.3. La medicación merma la capacidad para sentir las cosas con intensidad
	2.4. La medicación impide que las emociones se expandan
3. Experiencias con la medicación	3.1. La medicación ayuda a contener un impulso desbordante
	3.2. Experiencias de retirada y/o reducción de medicación. Vulnerabilidad y dependencia
	3.3. La evolución en el tiempo de la experiencia con la medicación
	3.4. La construcción de la experiencia con el hábito de tomar medicación
	3.5. La medicación es necesaria
4. Resistencia, superación, perseverancia: la persona	4.1. Resistencia biológica y antropológica
	4.2. La medicación transforma la personalidad, pero no la persona
	4.3. A la conquista de la calma interior
5. Relación con el sistema de salud mental	5.1. El dogmatismo de los profesionales de la salud mental
	5.2. Falta de información sobre la medicación
	5.3. Otros discursos identificados sobre la relación con los profesionales de la salud mental
6. Medicación, sociedad y comunidades	6.1. Medicación y control social
	6.2. El estigma social y comunitario con la medicación psiquiátrica

Fuente: Elaboración propia.

Como paso final del análisis, los investigadores ordenaron y relacionaron entre sí las categorías identificadas por los participantes, añadiendo una mayor interpretación. 

Todos los participantes participaron voluntariamente y firmaron un consentimiento informado, tanto para la reproducción y representación de fotografías como para las grabaciones. La resolución del Comité de Ética de la Universidad de Alicante fue favorable y recibió el código UA-2019-10-02.

## RESULTADOS

Los participantes fueron 11 personas (7 hombres y 4 mujeres) con una mediana de edad de 53 años, todos ellos sin un trabajo activo en el momento del estudio. Todos declararon una alta adherencia a sus tratamientos psicofarmacológicos, superior al 90% según el test de Morisky-Green^33^. La mediana de edad a la que empezaron a tomar por primera vez un tratamiento psiquiátrico crónico fue con 20 años (entre los 8 y los 25 años). Cinco de ellos vivían solos, tres de ellos lo hacían juntos a sus familias de origen y el resto convivían con pareja e hijos.

La Tabla 1 muestra las seis categorías identificadas con sus correspondientes subcategorías.

### Categoría 1. Cambio radical de vida a partir de la medicación

Esta categoría muestra cómo el inicio del proceso psiquiátrico (diagnóstico de trastorno mental grave e instauración del tratamiento psicofarmacológico) coincide con otras experiencias vitales significativas (crisis vital, nacimientos, muertes, rupturas…), conformando, todo ello, un conjunto global de vivencias, un acontecimiento radical que cambia la vida. La medicación se vive como un proceso irreversible, similar a un ascensor sin retorno, sin posibilidad de vuelta atrás. Por ejemplo, un participante (P2) realizó una fotografía en la que se veía un ascensor con un cartel rojo que decía en letras grandes: “NO USAR EL ASCENSOR EN CASO DE INCENDIO” y dijo lo siguiente:

“…*la medicación que tomamos no es un juego,* […] *no es un juego la medicación. La medicación es como subirte al ascensor… que ya vas a tomar la medicación para siempre, y te subes al ascensor*”. (P2)

Esto generaba una vivencia en la que se sentían “*encerrados*” (P2), en una “*cárcel*” (P1), “*de por vida*” (P2).

*“Tienes que estar tomándote la medicación, porque si dejas de tomar la medicación, esa puerta* [que aparece semiabierta en otra fotografía de P2] *se puede cerrar… y tienes que estar encerrado hasta que te recuperes otra vez. O a lo mejor no te recuperas y estás ahí de por vida”.* (P2)

*“Antes era libre como las gaviotas y ahora estoy en la cárcel. En el laberinto con las pastillas. Sin las pastillas no puedo pasar”.* (P1)

Además, se experimentaba una gran frustración por el “*precio*” que la medicación había supuesto en sus vidas. Por ejemplo, un participante al que le dijeron que la medicación le haría estéril, fotografió un parque infantil sin niños: “*es un parque que está vacío, y tendría que estar lleno*” (P2). Para algunos participantes la medicación simbolizaba la caída del paraíso, su vida anterior a la medicación, de la que guardaban un recuerdo idealizado, de “inocencia”:

“*…esa subjetividad crea un recuerdo de inocencia. Los recuerdos antiguos, de antes de nuestra… de nuestra enfermedad, ¿no? Queda un recuerdo de esas vivencias y de que éramos inocentes, que no sabíamos que… no conocíamos la enfermedad y todo lo que transmite esto…”.* (P7)

**Figura 1:**
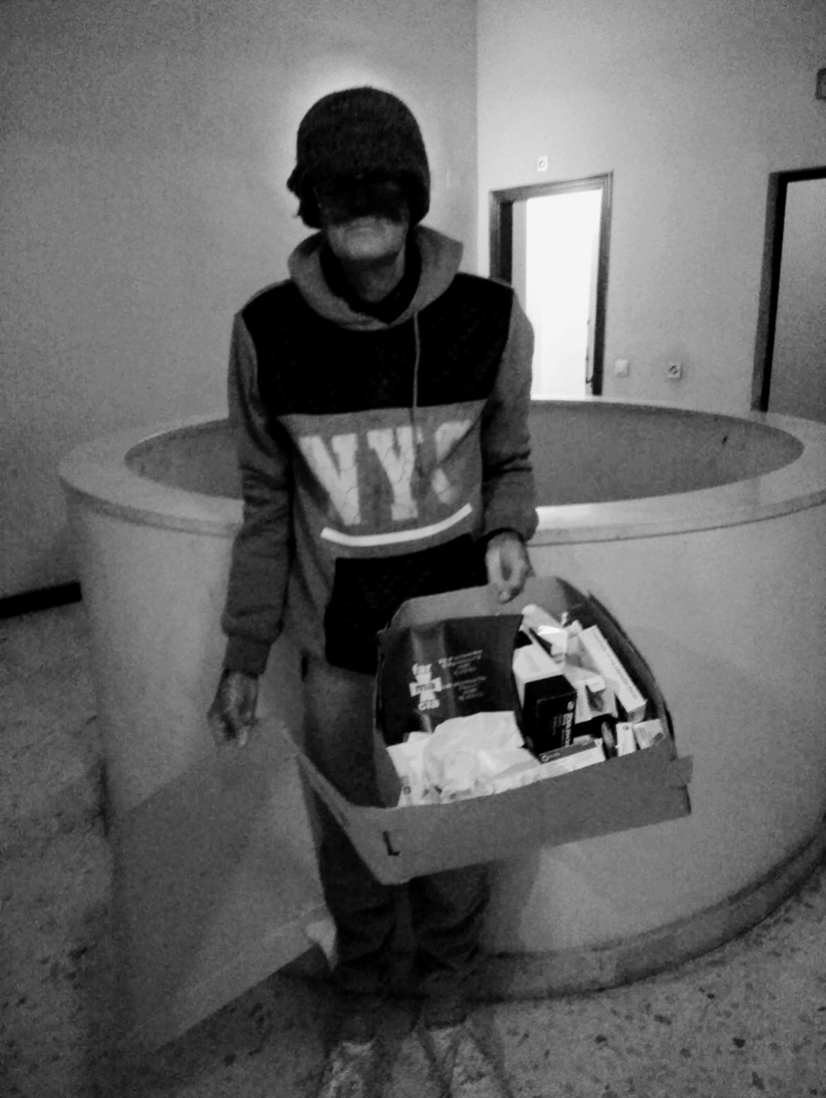
Halo de poder.

Las personas participantes expresaron que, con la medicación, acababan adoptando formas de vida más sedentarias puesto que, empezar el día, les suponía un desafío al arrastrar “*una carga de cansancio, con el peso de la medicación en las manos*” (Figura 1).

Fuente: Fotografía tomada por participante del estudio fotovoz, realizado en una escuela comunitaria dentro de un barrio de alto riesgo de exclusión social de una ciudad del sureste de España, 2018.

### Categoría 2. Cambios en la percepción, el pensamiento y/o el afecto

**Figura 2:**
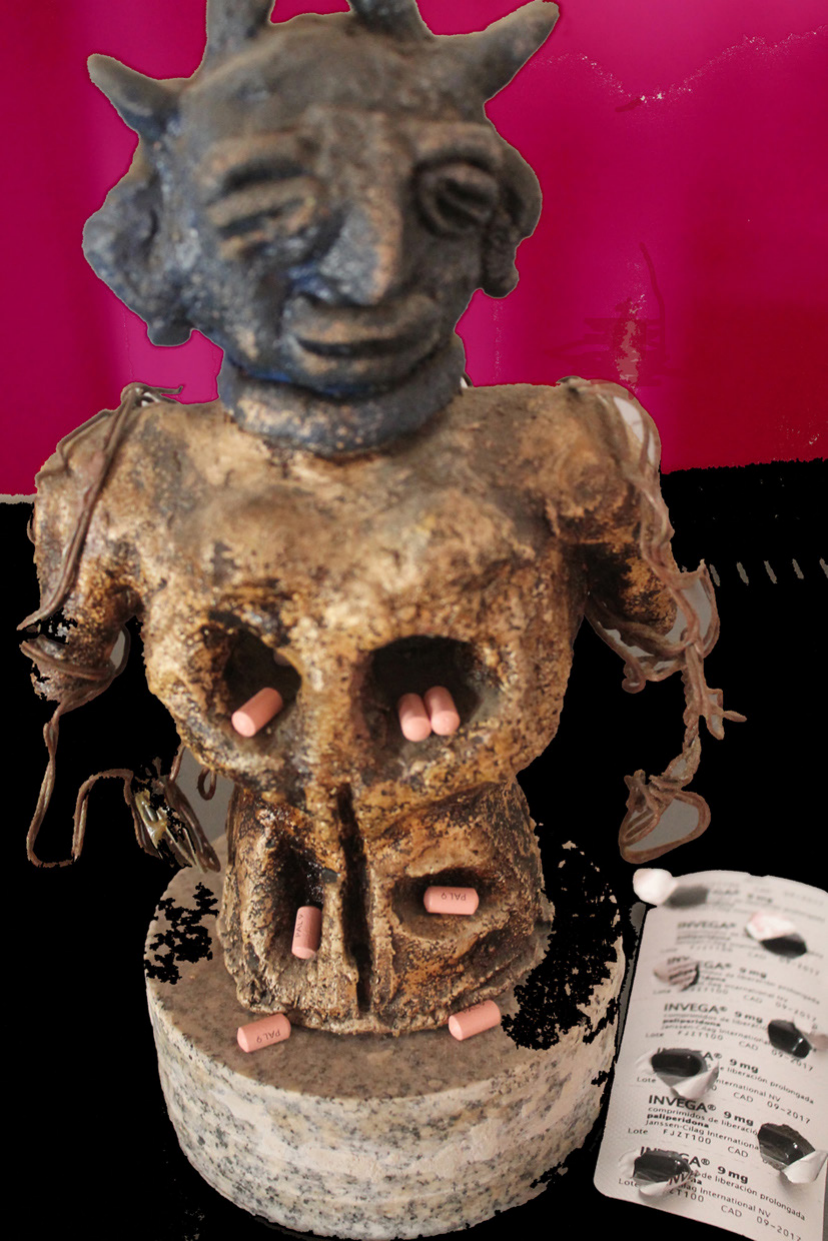
Vacío.

Una de las sensaciones cenestésicas asociadas a la medicación fue la de la intromisión en el cuerpo de un agente extraño cuyos efectos reales les son desconocidos y no controlados: “*te estás metiendo algo en el cuerpo que tú no sabes exactamente lo que es ni que efectos te va a hacer*”. (P4) (Figura 2).

Fuente: Fotografía tomada por participante del estudio fotovoz, realizado en una escuela comunitaria dentro de un barrio de alto riesgo de exclusión social de una ciudad del sureste de España, 2018.

**Figura 3:**
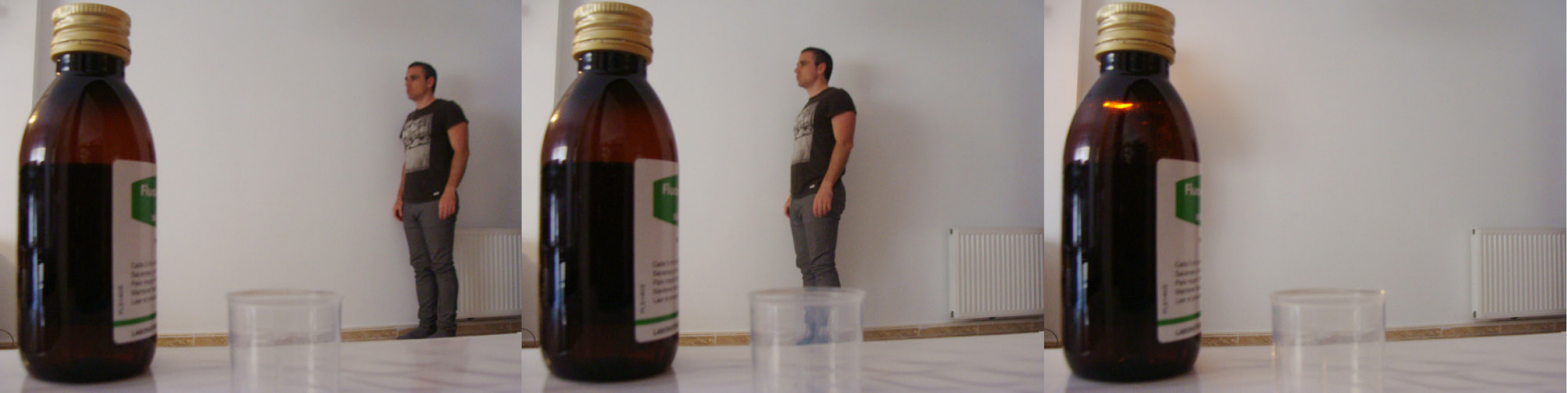
Identidad.

Otro aspecto destacable fue la manera en que la medicación influía en la percepción de sí-mismo, y en la identidad. Uno de los participantes en tratamiento con fluoxetina, hizo una serie secuencial de tres fotografías (Figura 3) en la que él mismo iba aproximándose hacia la medicación hasta que su figura desaparecía absorbida por el frasco. En la última fotografía de la serie solo se veía el frasco de cristal topacio al fondo del cual emergía una luz “*para no perderme del todo en el frasco de medicación*” (P3). Esa luz pequeña pero intensa representaba a un participante sin forma absorbido en el líquido farmacológico.

Fuente: Fotografía tomada por participante del estudio fotovoz, realizado en una escuela comunitaria dentro de un barrio de alto riesgo de exclusión social de una ciudad del sureste de España, 2018.

Uno de los conflictos que expresaron fue el sentir que la medicación los anestesiaba, de manera que no podían “*sentir con intensidad las cosas*” (P4). Por ejemplo, uno de los participantes contó que ante la muerte de su amigo “*necesitaba llorar y no podía*” (P7), porque la medicación no le permitía expandir esa emoción; esto le generaba una “*fuerte inquietud*”. Otro participante refirió que la creatividad artística era fundamental para su vida, pero que el medicamento que tomaba (paliperidona) se la “*borraba por completo*”:

“*Yo pintaba desde hace mucho tiempo…hacía fotografías …y, hacía cosas creativas…tenía un cierto sentimiento de melancolía casi siempre dentro de mí, que eso…el medicamento me lo ha borrado por completo. O sea, el medicamento me ha cortado la capacidad que tenía de sentir las cosas y me ha puesto en una situación más normal, más anodina*”. (P4)

Para ellos, la implicación de esta limitación era enorme, pues percibían la creatividad como algo a lo que estaban predestinados “*estamos destinados a ser creativos* […] *tenemos una disposición a crear*” (P7). Uno de los participantes llegó a confundir “creatividad” con “enfermedad”, como si fueran dos caras de la misma moneda.

### Categoría 3. Experiencias con la medicación

**Figura 4:**
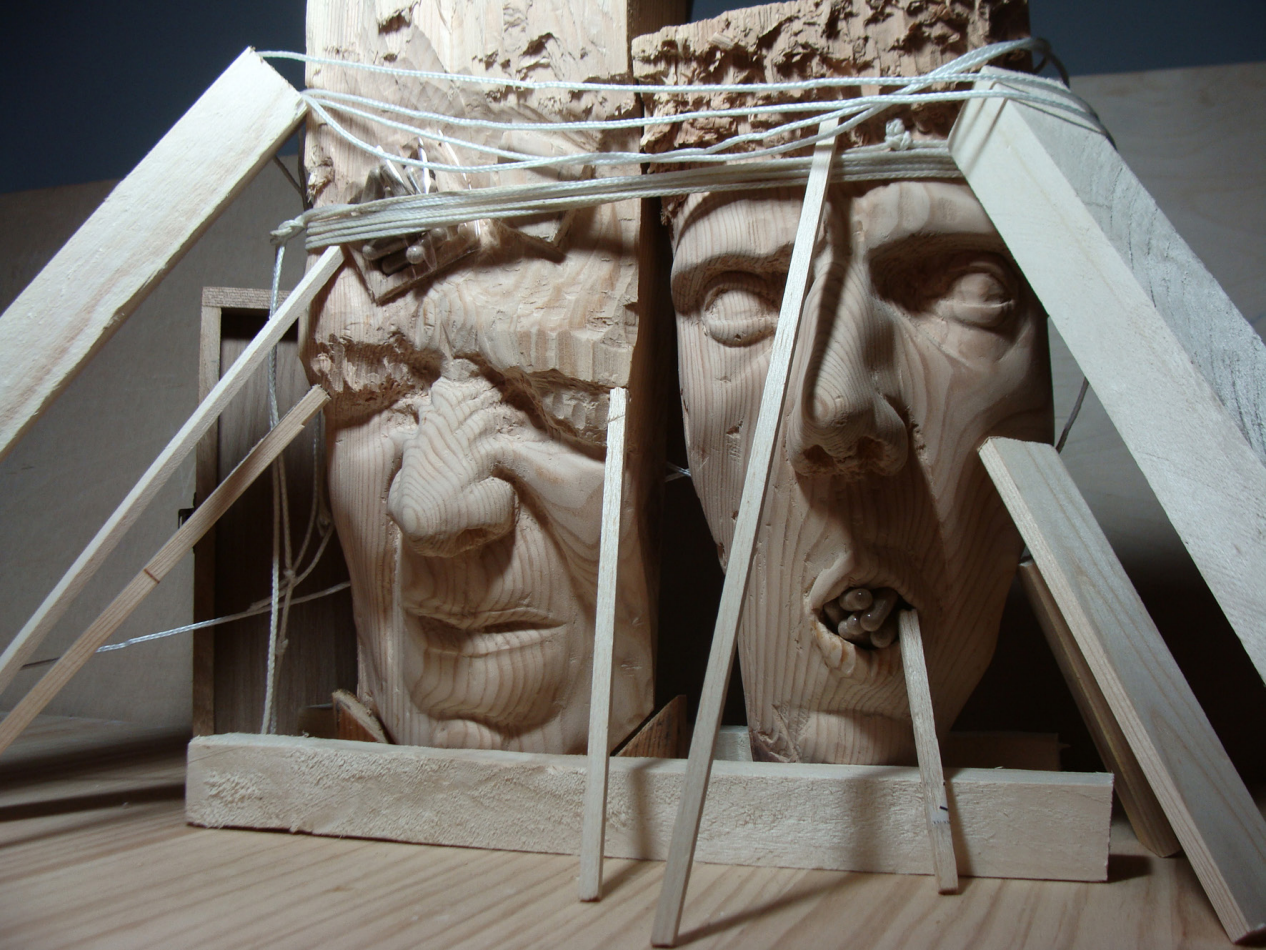
Equilibrio precario.

Las personas participantes expresaron cómo la medicación les ayudaba a contener “*impulsos desbordantes*”. En una de las fotografías (Figura 4) la medicación se representó con unas barras de madera que sostenían a todo un sistema-vida (las cabezas esculpidas) que estaría a punto de derrumbarse. Las esculturas no podrían sostenerse por sí mismas pues emergería una “*ansiedad y pensamientos negativos descontrolados*” (P3), de naturaleza autodestructiva, que tendían a derrumbar el conjunto. La medicación vendría a “*frenar o contener, apuntalar, todos esos pensamientos negativos, todos esos impulsos*” (P3), como un elemento que mantendría la “caja de Pandora” cerrada; haría posible, por tanto, que el sistema-vida se sostuviese, aunque fuera en un estado de “*equilibrio precario, porque apenas se sostienen*” (P3). 

Fuente: Fotografía tomada por participante del estudio fotovoz, realizado en una escuela comunitaria dentro de un barrio de alto riesgo de exclusión social de una ciudad del sureste de España, 2018.

En relación con esto, cualquier propuesta de modificación en la psicofarmacoterapia (dosis, forma farmacéutica, marca comercial, fármaco o aspecto) podría vivirse como una alteración de este “equilibrio precario” y, por tanto, con vulnerabilidad y temor. 

Respecto a la evolución en el tiempo de la experiencia con la medicación, algunas personas expresaron haber encontrado una “*química perfecta*” tras un camino de ensayo-error. El hecho de tener que cumplir con un horario de administraciones era vivido por algunos participantes con estrés; mientras, para otros, lo principal era el aburrimiento. Una participante fotografió una agenda semanal que contenía actividades lúdicas (circo, natación…) y otros quehaceres (recoger la ropa…), pero una anotación en rojo gobernaba todo el cuadro del tiempo: “*Tomar medicación*”. La costumbre acababa por disolver el conflicto, normalizando la experiencia de cumplir con el tratamiento. Paralelamente, se iba construyendo una asociación entre sentirse bien y la toma de medicación. Y, al contrario, entre “*estar mal*” y no haber tomado la dosis todavía, lo cual generaba la narrativa de que, en última instancia, la medicación les era necesaria.

Como parte del discurso de que *“La medicación es necesaria”,* una de las participantes manifestó de manera rotunda que la medicación que llevaba veinte años tomando no le había “*quitado nada*” (P10) sino que, al contrario, le había permitido llevar una vida satisfactoria, aportándole funcionalidad en su día a día y fomentando su socialización. En su proceso de recuperación, uno de sus principales apoyos había sido formar parte de una red de cuidados y afecto (amigos y familia), aunque “*lo principal yo creo que es la medicación*” (P10). Para otro de los participantes, la medicación acababa siendo una “*necesidad fisiológica, como el comer, o el beber, o hacer el amor*” (P10); la medicación psiquiátrica era “*para los enfermos mentales, como la insulina para los diabéticos*” (P10):

“*La medicación tiene sustancias que van bien al cuerpo* […] *como un diabético que está con su insulina* […] *o sea, una persona que se pincha insulina está estable* […] *Me refiero a eso. Una persona no se para: ‘Uy, soy diabético y tal’ … ¡es diabético y ya está!, ¡se tiene que poner insulina!*”. (P10)

Esta conceptualización de la enfermedad mental como una diabetes y, por tanto, la analogía de la medicación psiquiátrica con la insulina implicaría una asociación implícita entre medicación psiquiátrica y trastorno mental, de manera que la carencia de medicación supondría un empeoramiento de la salud mental y, a la inversa, una mejoría conllevaría su toma ininterrumpida.

“*Si no te medicas vas a peor, si te medicas vas a mejor. O sea, eso es lo principal. Yo no me medico por gusto, me medico por una enfermedad mental*”. (P10)

“*La necesidad de tomarla es la obligación de no dejarla de tomar nunca. Porque la medicación es para los enfermos… uno que está bien no toma medicación*”. (P5)

“*Si te quitan la medicación es porque estás mejor de tu enfermedad*”. (P9)

**Figura 5:**
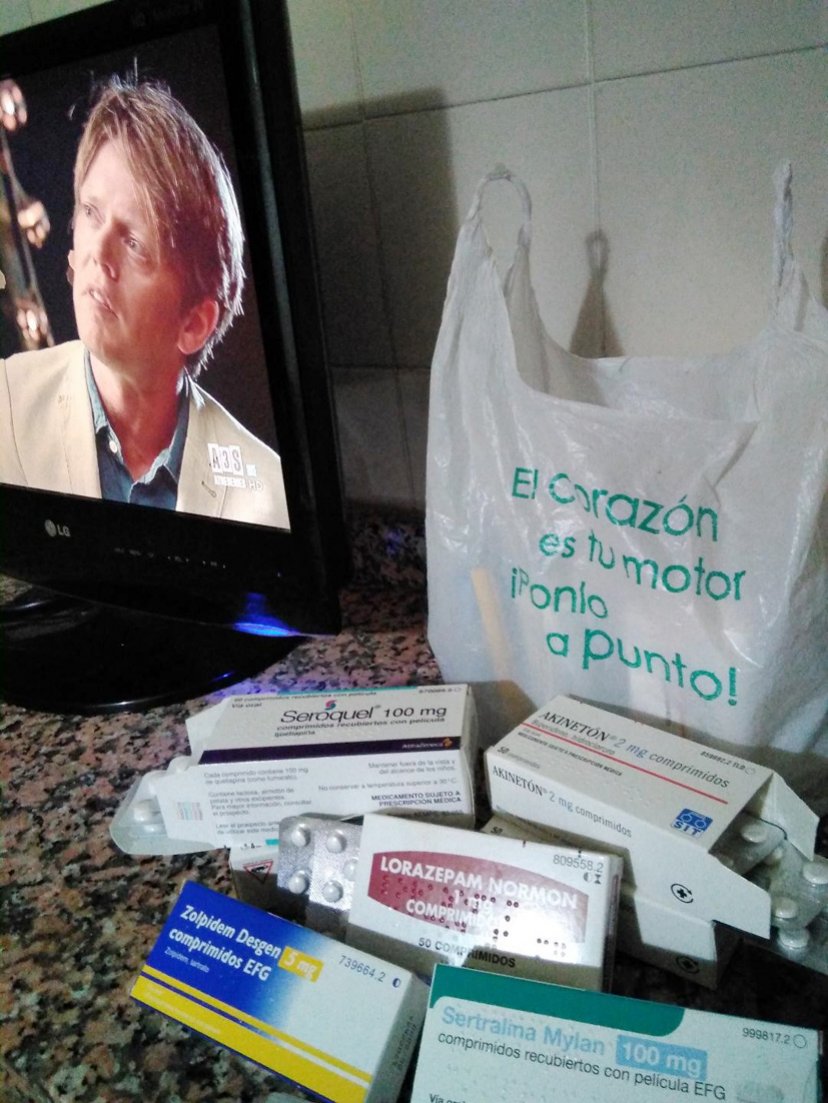
Normalidad.

Así, la analogía entre la medicación psiquiátrica y la insulina contribuiría a una vivencia de “normalidad” (Figura 5) y ausencia de conflicto.

Fuente: Fotografía tomada por participante del estudio fotovoz, realizado en una escuela comunitaria dentro de un barrio de alto riesgo de exclusión social de una ciudad del sureste de España, 2018.

### Categoría 4. Resistencia, superación, perseverancia: la persona.

Emergió la noción de una resistencia como “*defensas*”, “*anticuerpos*”, “*sustancias*” que “*rebaten la química que produce la medicación*” (P7), como una resistencia biológica que se contraponía a las adversidades introducidas por la medicación. De esta forma, el paciente no sería un sujeto que padece o sufre los efectos inevitables de una medicación, sino alguien que puede construir activamente el resultado final de los efectos farmacológicos:

“*Cuando una medicación te hace bien, o te hace mal, independientemente, queda la sustancia, y esa sustancia, tú combates esa sustancia, y tienes anticuerpos, tienes defensas. En el cerebro también hay sustancias, que rebaten esa química que produce la medicación* […] *cuando la medicación no es adecuada, tú la combates. La mente crea sustancias que se oponen a la medicación*”. (P7)

Explicaron que la medicación produce efectos adversos que pueden ser difíciles de vivenciar y que la voluntad es lo que permitiría sobreponerse a ellos. A eso, un participante lo llamó “*creatividad*” (P7), a ese “*alzarse por encima de tus posibilidades*” (P7) a ese “*elevarse*” para sobreponerse a la “*carga*” (P3), del “*peso*” (P3 y P7) que los efectos de la medicación pudieran provocar.

“*Yo también me doy cuenta… de que lo que yo hablaba era que la medicación produce un malestar, hablaba de eso. Y yo lo que creo también es que, si tienes la voluntad suficiente para producir más de lo que eres capaz, eso es creatividad. Cuando te vales por encima de tus posibilidades, eso es el hombre que vale más. Hay veces que el ser humano se eleva por encima de sus posibilidades, y no siempre, pero hay situaciones y momentos en los que el ser humano es capaz de hacerlo mejor* […] *y la mente se levanta por encima, de tal manera que se sobrepone a ese peso agobiante*…”. (P7)

**Figura 6:**
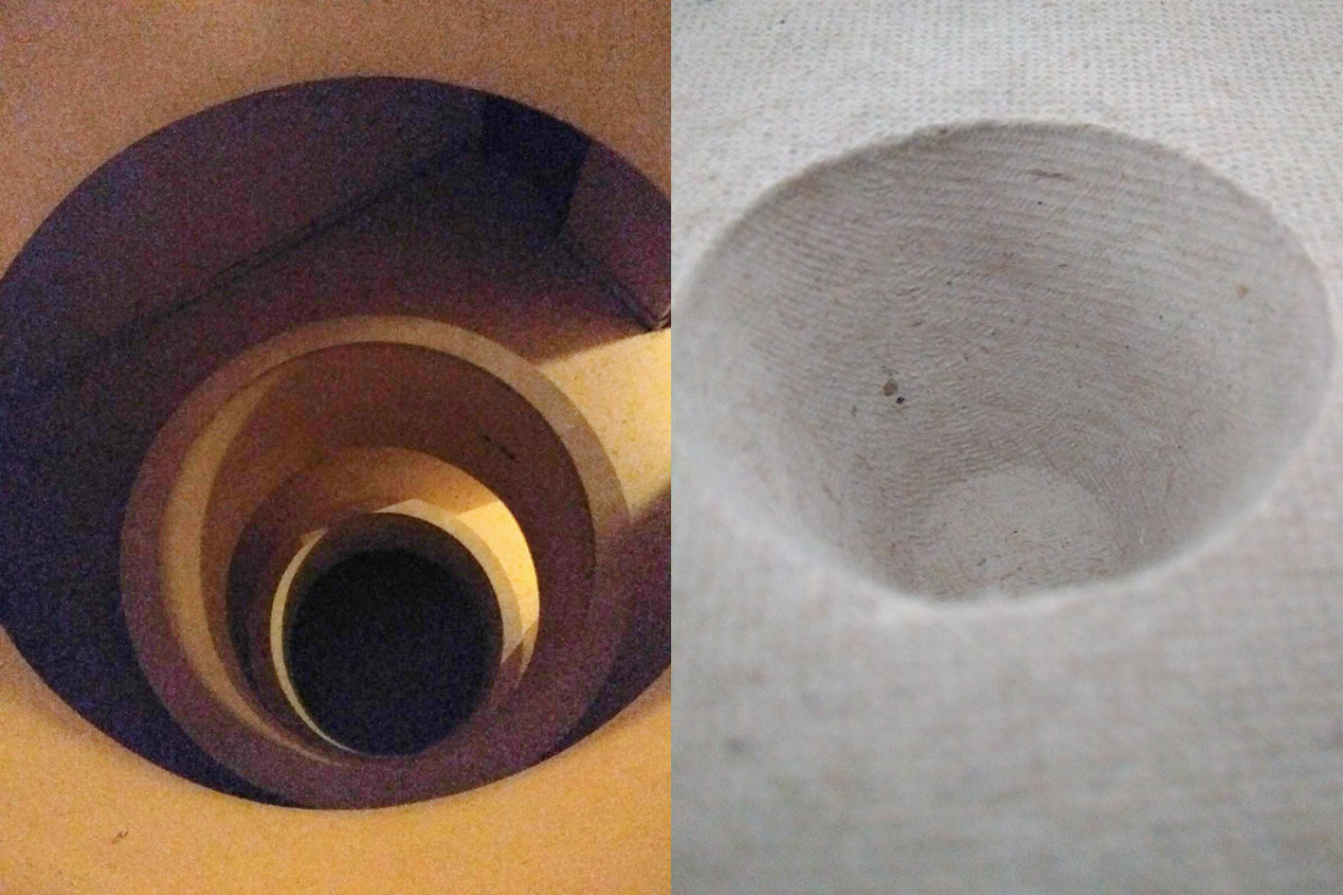
a) El laberinto de la vida y la muerte; b) De aquí se sale.

Así, al fondo de ese “Laberinto de la vida y la muerte” (parte a de la Figura 6) con el que uno de los participantes representaba su vida con medicación, no solo habría oscuridad, sino también un camino de resistencia, una manera de perseverar en lo mejor de uno mismo, esfuerzo (voluntad, valor y dignidad que se alzan frente a la carga farmacológica), representada en otra de las fotografías del mismo autor (parte b de la Figura 6) y que viene a ser la otra cara del laberinto, donde el agujero negro se transforma en un agujero blanco en conexión profunda con el anterior y que lleva por título “De aquí se sale”.

“*Por ejemplo… yo me he despertado hoy desorientado… he dormido bastante y me he despertado desorientado, sin saber dónde estoy, dónde me ubico, dónde empezaba mi día mentalmente… por eso la mentalización es muy importante. Puede jugar un papel importante en alguno de estos criterios* [refiriéndose a las categorías que estaban emergiendo]… *Mentalización no solo cuando te despiertas y estás desorientado y no sabes cómo colocarte mentalmente para preparar el día en tu mente y en tu vida y organizarte, sino la mentalización más allá, en cualquier horario del día. Y eso te invade y puede dar una respuesta a tu inquietud. Añadir algo a tus pensamientos y a tu forma de ver la vida*”. (P7)

Fuente: Fotografía tomada por participante del estudio fotovoz, realizado en una escuela comunitaria dentro de un barrio de alto riesgo de exclusión social de una ciudad del sureste de España, 2018.

El grupo matizó que “la medicación transforma la personalidad, pero no la persona” (P7). De nuevo emergía la idea anterior: había un ser activo que permanecía intacto: “yo soy el maestro de mi propio idioma […] la persona sigue siendo íntegra” (P7). Este ser activo guiaba al sujeto, y lo orientaba: “se pueden cultivar nuevos sentimientos, nuevas capacidades” (P7). 

Los participantes manifestaron que buscaban alcanzar estados de calma interior fundamentalmente, a través de prácticas artísticas (como el dibujo), y del contacto con la naturaleza: “*la naturaleza es algo vivo*”. Esta contemplación de la naturaleza se describía como una fuente de conexión con uno mismo en una especie de meditación interior. Los participantes relacionaron a los animales domésticos como parte de esta naturaleza y relataron experiencias de recuperación-curación gracias al vínculo y comunicación con ellos.

“*Yo he visto una ahí con dos gatos que me parece muy interesante… lo de las mascotas y la... y, la curación. Entre la curación y las mascotas…o sea que un perro o un gato te pueda ayudar a… a existir*”. (P1)

### Categoría 5. Relación con el sistema de salud mental

Los participantes se quejaron de no haber recibido información suficiente acerca de los tratamientos farmacológicos: “*de haberlo sabido, me lo hubiera pensado sin duda*” (P3); de que se pusiera el énfasis del tratamiento en atacar al síntoma en lugar de la consecución de una salud global; y de que, en ocasiones, los juicios emitidos por los profesionales sanitarios hubieran sido demasiado dogmáticos. Algunos valoraron positivamente la actitud de sus psiquiatras a la hora de explicar dudas y de respetar sus decisiones, mientras que otros, calificaron la actitud de estos profesionales de “*prepotencia*” (P4). Refirieron que, en ocasiones, tanto sus psiquiatras como otros profesionales de la salud mental habían emitido juicios, mensajes o enunciados “*muy drásticos*” (P10), “*categóricos y definitivos*” o “*muy mal enfocados*” (P7).

“*Cuando yo entré en ese centro* [psiquiátrico] […] *yo no sabía si era un reformatorio, una cárcel… y me dijeron: ‘de aquí no vas a salir’ …yo tenía 18 años*”. (P10)

La relación que manifestaron con el sistema de salud era, fundamentalmente, a través del psiquiatra. Sobre esta figura se identificaron dos tipos de discurso: uno que tenía como núcleo terapéutico la toma de medicamentos y que estaba ligado a la idea de “*obligación*” de tomarlos para toda la vida:

P3: -“…*el eje fundamental del proceso terapéutico es la toma de fármacos* […] *la medicación es para toda la vida”.*

P5: -“*Pues… paciente-enfermo… obligación de mantener una medicación siempre*”.

Investigador: -“*A ver, por ejemplo, ¿tú cómo llamarías a la relación con tu psiquiatra?*”

Y otro discurso más parecido a un modelo de toma de decisiones compartidas donde sus psiquiatras les respondían “*con naturalidad*” y les ofrecía soluciones satisfactorias a sus dudas y conflictos.

### Categoría 6. Medicación, sociedad y comunidades

El grupo refirió que, con frecuencia, sufrían la deslegitimación de estados que podían ser normales e incluso necesarios (como el enfado, la rabia, o la rebeldía) por estar diagnosticados de un trastorno mental o por el hecho de estar medicados: “*Si estás enfadado es porque no te has tomado la pastilla*” o “*¡anda, tómate la pastilla!*” (P10) (en el contexto de un conflicto). Pero si lo que subyacía al conflicto era, por ejemplo, un abuso de poder (por ejemplo, en el caso de una mujer que en su casa se sobrecargaba con tareas del hogar y se enfadaba con su marido que no hacía nada), al final, lo que ocurría es que dicho abuso y control se perpetuaba. El medicado salía desfavorecido con respecto al no-medicado, pues sus reacciones emocionales ante los conflictos eran interpretadas como parte de una enfermedad, o de la medicación o de la falta de la misma, y no porque hubiera algo de legítimo en ellos.

“*Eh… yo llevo un par de semanas tomando medio Seroquel. Tomaba antes uno a la noche. Y… en cosas que antes no me molestaban: ‘pues vale, no pasa nada’, yo ahora me quejo, y al quejarme me dicen: ‘Tal. Tendrás que tomarte media pastilla más…’. Y yo les digo: ‘No. No’. Pero cosas que antes me daban igual, que yo decía: ‘sí, no te preocupes que yo…’ ahora digo: ‘no quiero’* […] *Yo me refiero a una cosa que tú haces normal, por ejemplo, bajar todos los días a por el pan, tal… y un día, pues… no quieres bajar. No bajo porque no quiero. ¡Baja tú! Es decir, eso simplemente, a mi marido le…* [incomprensible]… *y… que me diga eso de ‘tendrás que tomarte media pastilla más…*’ *¡pues no!*”. (P10)

A la inversa, si en un conflicto relacional la persona medicada mostraba valores como la templanza, la reflexión o la contención del enfado, algunos participantes sentían que eran méritos propios que acababan siendo devaluados por el no-medicado, atribuyendo esos méritos a la medicación. Así, el mostrarse apacible y sosegado en medio de un conflicto, era vivido como una virtud cultivada y, sin embargo, la persona no-medicada co-implicada en el conflicto, atribuía esa calma o sosiego a los efectos de la medicación, devaluando el mérito que el participante pudiera realmente tener. Como el propio sujeto recalcó: “*yo no soy excesivamente apacible por tomarme la medicación, sino… porque yo he cultivado ser apacible*” (P7).

La sensación que generaba adquirir un diagnóstico de trastorno mental y/o recibir medicación psiquiátrica era descrita como un «no poder escapar de la etiqueta impuesta». Una vez adquirida la etiqueta, se sufría un proceso de clasificación y división del resto de la sociedad a través de estereotipos que acababan convirtiéndose en verdades:

“*Si nosotros consideramos que estamos enfermos están creando una verdad aparente. Estamos creando una cosa que cohabita. Si nosotros nos lo creemos, estamos creando una verdad. Esa verdad se traduce a los demás. No puedes escapar de esa realidad, porque si tú no estás enfermo, no puedes considerarte una persona enferma. Es que, la verdad tiene una fuerza increíble*”. (P7)

Algunos participantes refirieron tener pánico a que la gente supiese que se encontraban bajo tratamiento psiquiátrico y sentían una enorme necesidad de mantenerlo en secreto ante el miedo a ser estigmatizados por ello y ser expulsados tanto de la sociedad (falta de empleo y acceso a recursos básicos) como de la comunidad (expulsión de grupos y/o relaciones afectivas).

## DISCUSIÓN

Los resultados de este estudio muestran que los medicamentos funcionan, además de como moléculas farmacológicamente activas, como símbolos que ejercen un campo de acción práctica en las personas que conviven con ellos. Este elemento simbólico forma parte de un proceso de construcción de significado marcado fuertemente por la experiencia farmacoterapéutica y que determina, en última instancia, la actitud y adherencia hacia la medicación. Todo esto manifiesta la clásica definición griega de qué es un fármaco. *Pharmakón* es, al mismo tiempo, lo que te puede curar y lo que te puede matar, remedio y veneno, por lo que el conflicto ya está impreso en la ontología del fármaco^34^. Del mismo modo, las metáforas empleadas por el movimiento social “Orgullo Loco”, analizadas en un estudio lingüístico, conceptualizan sus vidas mayoritariamente en términos de “guerra” y “conflicto”^32^.

Las vivencias identificadas en el presente estudio pueden desglosarse en dos grandes núcleos simbólicos: uno de conflictividad y otro motivacional hacia el tratamiento. El núcleo de conflictividad está marcado, a su vez, por dos grandes elementos: a) la medicación como símbolo de una caída en una vida marcada por el trastorno mental-proceso psiquiátrico y b) la medicación, como símbolo de la vulnerabilidad, fragilidad y falibilidad. En cuanto al núcleo motivacional, se identifican tres elementos simbólicos: a) la búsqueda de un alivio inmediato para la angustia, b) el discurso que manejan las instituciones de salud mental basado en una especie de “tratamiento neurotransmisor sustitutivo”, en analogía con la insulina en diabetes tipo I (aunque esta narrativa puede virar hacia el primer núcleo simbólico de conflictividad y coacción en las personas y colectivos que deciden no incorporarlo), y c) resistencias, tanto las biológicas (neuroadaptación compensatoria hacia los efectos directos de los fármacos), como una resistencia antropológica y moral. 

El “discurso de las instituciones” sería aquel que el dispositivo institucional articula con el objetivo de investigar, comercializar, motivar y, con frecuencia, coaccionar hacia la toma crónica de psicofármacos^35^^,^^36^^,^^37^ y se nutre del biologicista hegemónico en medicina clínica y en psiquiatría^38^^,^^39^^,^^40^. Establece una analogía de los trastornos mentales con el modelo de enfermedad de la diabetes mellitus tipo I^41^. La cadena, que va desde el desequilibrio biológico (carencia bioquímica que se suple farmacológicamente) hasta el resultado clínico y de morbimortalidad, funcionaría como una perfecta máquina mecánica sustentada en una metafísica cartesiana^42^. El fármaco vendría a suplir la pieza que falta en el puzle y con ello conseguiría el restablecimiento de todo el sistema bio-psico-social, convirtiéndose así en la pieza nuclear de todo el sistema terapéutico, irremplazable y necesario para la viabilidad del paciente. Este modelo es además el que mejor se acoplaría al sistema mundial de investigación-comercialización de medicamentos^36^^,^^38^^,^^39^^,^^43^, por lo que el discurso sirve, en última instancia, a la concepción del fármaco en tanto mercancía^37^^,^^44^^,^^45^*.* Es decir, este discurso se acopla de forma precisa y eficiente al sistema de producción mundial de medicamentos, haciendo que el fármaco funcione como mercancía en el mercado de la industria de la disfunción (en teoría del valor, extracción de un plusvalor). El hecho de que pueda, además, acabar funcionando como remedio (eventual extracción de su valor de uso), es un hecho accidental que depende de que personas, colectivos y profesionales nos volquemos performativa y colectivamente hacia tal proceso. Cabe notar que este discurso-modelo funciona con toda la potencia dogmática propia de un discurso ultra-elaborado y acabado, y de ahí que las vivencias generadas en los participantes hagan alusión a ello: mensajes categóricos, muy drásticos, con prepotencia, que tienden a clausurar procesos dialógicos y epistémicos en aquellos que no se “tragan” estas narrativas (ni las pastillas que las acompañan). El concepto de adherencia se introdujo para distanciarse del “incumplimiento”, como un intento de pasar de una cuestión de obediencia y autoritarismo a una cuestión ética, dialógica y multicultural^46^. Para terminar, según se reconoce en el informe del Relator Especial de Naciones Unidas, este discurso es pseudocientífico y actúa con carácter trascendental, como condición de posibilidad de violencia institucional. Efectivamente, la epistemología biologicista y la violencia institucional comparten una misma raíz metafísica^42^.

La vulnerabilidad y fragilidad se revive especialmente en los procesos de retirada y/o disminución de carga psicofarmacológica. Montcrieff critica que las reducciones, cuando se practican, no tengan en cuenta los procesos de neuroadaptación que se instauran con el tratamiento y que es necesario revertir con retiradas muy progresivas^47^^,^^48^^,^^49^. Pero esto no solo tendría un sentido biológico, sino también un sentido antropológico, como se han mostrado los resultados de este y otros estudios^50^. 

La medicación puede significar una forma de control en el seno de conflictos interhumanos, y puede verse, en última instancia, como el pasaporte definitivo (al igual que el diagnóstico) hacia procesos de estigmatización^25^^,^^29^^,^^51^^,^^52^. La medicación podría contribuir al estigma de dos formas: por un lado, por la concepción sociocultural degradada que se tiene de la medicación psiquiátrica^52^ y, por otro lado, por algunos efectos puramente farmacológicos (que se reflejan en la expresividad y gesticulación, en el habla o en el cuerpo)^12^. El miedo a que los demás descubran que se está bajo tratamiento psiquiátrico desgarra el proyecto de vida que queda como parte del paraíso perdido, pendiente de rehabilitar. A su vez, las personas que sufren estigma, son propensas a abandonar el tratamiento y/o mantener bajos grados de adherencia^25^^,^^52^, lo cual podría reforzar la idea de que la medicación queda impregnada de la fuerza simbólica de “caída” en el trastorno mental (si dejo la medicación, salgo del proceso psiquiátrico). 

En este estudio se han identificado dos tipos de resistencias: resistencias a nivel biológico (neuroadaptación) y resistencias a nivel antropológico (la persona). Por una parte, se alude a procesos de neuroadaptación (homóloga y heteróloga), que los efectos inmediatos de los psicofármacos inducirían en el sistema nervioso central con el tiempo^47^^,^^53^, como los clásicos procesos de tolerancia farmacológica. Es algo bien conocido con los opiáceos o benzodiazepinas pero que también acontece con antipsicóticos o antidepresivos^47^^,^^48^^,^^54^^,^^55^. Estos fenómenos explicarían la tendencia al aumento acumulativo de carga psicofarmacológica prescritas en personas diagnosticadas de trastorno mental grave^56^. Este rasgo bio-farmacológico deja, por tanto, un rastro vivencial, una constatación fenomenológica de que el cuerpo resiste desde el cuerpo. Por otra, según los participantes, la persona se conserva “al fondo” del proceso psiquiátrico; una instancia sintiente que permanece al fondo de los padecimientos y acciones de la tríada vida-trastorno mental-medicación. 

Hay una manifiesta conexión entre los elementos emergidos en las experiencias de gestión autónoma de la medicación en Brasil y Cataluña, plasmadas en la “Guía para la gestión colaborativa de salud mental” y los resultados de nuestro estudio: experiencias con la medicación, dimensión simbólica del medicamento y su entrelazamiento íntimo con la red social, las instituciones y sus profesionales, los procesos de estigmatización y la pobre o rica calidad de los procesos de toma de decisiones compartidas^29^. Asimismo, hay elementos narrativos comunes con otros estudios cualitativos llevados a cabo en Chile y España. El análisis lingüístico publicado en 2020^32^, que emerge a partir de la manifestación del “Orgullo Loco”, pone de manifiesto metáforas que identifican experiencias de ingreso psiquiátrico en tanto “tortura” violenta, en las que no hay diálogo, sino involuntariedad y coacción. El primer contacto de las personas con la psiquiatría (diagnóstico e inicio de tratamiento), que en nuestro estudio se calificó de “acontecimiento radical”, en este estudio se conceptualizó como una “fractura biográfica”, “un antes y un después” que crea una “discontinuidad con la vida anterior”. En cuanto al estudio chileno publicado en 2018^50^, también pone de manifiesto el íntimo entrelazamiento de los efectos farmacológicos y síndromes psiquiátricos con las experiencias vitales (violencia familiar, divorcio, separación de hijos o hechos sociales). También se habla de “bloqueo mental”, anestesia o contención emocional y deterioro cognitivo asociado a la medicación y de vivencias de violencia y abuso en el sistema de salud mental. Cabe destacar las experiencias de vulnerabilidad y fragilidad que relataban sus participantes (al igual que los nuestros) cuando afrontaban retiradas o disminución de medicación.

También son evidentes algunos paralelismos con los estudios de fotovoz publicados en el mundo anglosajón^18^^,^^25^^,^^57^. Gran parte de nuestros resultados podrían integrarse en lo que sus autoras denominaron “aceptando la medicación”, proceso al cual remiten las motivaciones y conflictos en torno al tratamiento psiquiátrico. También se aludía a que la medicación lograba *contener* la sintomatología psiquiátrica y empleaban una descripción muy similar al símil de “caja de Pandora”. Sus participantes también manifestaban confusión e incertidumbre al no saber qué de lo que experimentaban se debía a un efecto secundario de la medicación y qué se debía a otras causas. Otros elementos narrativos en común eran la experiencia de agobio (de carga) al ver toda la medicación del pastillero, la importancia simbólica de los animales de compañía en el proceso terapéutico, el conflicto vivencial en torno al deterioro cognitivo, o la vivencia de que la medicación era indispensable en sus vidas. 

La medicación, en cualquier caso y según todos estos estudios, no es algo que pudiera ser *aislado fenomenológicamente* del proceso psiquiátrico y vital en su conjunto, lo que quiere decir que la vivencia de la medicación está íntimamente relacionada con la experiencia que estas personas tienen de sus síntomas, con el proceso psiquiátrico que han vivido y, en última instancia, con sus vidas. Así, la adherencia al tratamiento farmacológico es un proceso de *construcción de significado* dentro de este magma vital. Esto suscita preguntas importantes para el proceso farmacoterapéutico que marcan próximas líneas de investigación. La dimensión simbólica de la medicación podría ofrecer un marco para la comprensión del alto grado de efecto placebo encontrado en los ensayos clínicos. De asumir esta hipótesis, no sería adecuado presuponer que la psicofarmacoterapia va a ejercer su efecto biológico de forma autónoma, independientemente de la experiencia. Más adecuado sería asumir un modelo bio-antropológico, en el que la experiencia con la medicación modula la respuesta farmacológica, y a la inversa. Esto presenta enormes implicaciones. Por ejemplo, cómo funciona simbólicamente el tratamiento farmacológico en el contexto de los ingresos involuntarios o de medicación forzosa y si, en estos casos, el tratamiento puede de hecho generar vivencias significativas.

Según los criterios de calidad de investigación cualitativa descritos por Guba y Lincoln^58^, nuestros resultados reflejan una diversidad de narrativas con un grado de conflictividad variable. Contribuyó a la credibilidad de los resultados el hecho de que la sesión de categorización se realizase junto con los participantes. En cuanto a la transferibilidad, podrían encontrarse resultados similares dentro del perfil de participantes y el contexto de nuestro estudio. Contextos hospitalarios o institucionalizados o personas bajo un régimen de tratamiento ambulatorio involuntario no serían representativos de la experiencia analizada. En cuanto al criterio de no-transferibilidad habría que discutir sobre el grado de deterioro cognitivo. A pesar de que hubo variabilidad significativa en el nivel cognitivo entre los participantes, este no supuso una limitación en la representatividad de las narrativas. El hecho de que las narrativas emergiesen de una imagen artística pudo haber facilitado la expresión de personas con un mayor deterioro lingüístico, verificándose lo que otros autores señalan sobre la idoneidad del método de fotovoz en estas situaciones^19^^,^^20^^,^^21^. Con respecto a la confirmabilidad, pensamos que queda salvada por los siguientes factores: a) la propia narrativa de los participantes que, en todo momento, fue cuidada, respetada y fomentada por los facilitadores dialógicos; b) la diversidad situacional del equipo investigador en cuanto a género, formación y generación; c) la triangulación en la fase hermenéutica de resultados; y d) nuestro compromiso ético con el rigor epistémico y la ausencia explícita de ningún conflicto de interés.

## CONCLUSIÓN

Los resultados de este estudio muestran que los medicamentos funcionan, además de como moléculas farmacológicamente activas, como símbolos que ejercen un campo de acción práctica en las personas que conviven con ellos. Este elemento simbólico forma parte de un proceso de construcción de significado marcado fuertemente por la experiencia farmacoterapéutica y que determina la actitud y adherencia hacia la medicación. Las vivencias identificadas en el presente estudio pueden desglosarse en dos grandes núcleos simbólicos: uno de conflictividad y otro motivacional hacia la medicación psiquiátrica.
